# Applying narrative medicine to prepare empathetic healthcare providers in undergraduate pharmacy education in Singapore: a mixed methods study

**DOI:** 10.1186/s12909-024-05254-z

**Published:** 2024-03-15

**Authors:** Zhe Han, Keith C. Barton, Li-Ching Ho, Kai Zhen Yap, Doreen Su-Yin Tan, Shuh Shing Lee, Constance Xue Rui Neo, Amanda Han Lin Tan, Brian Ming Yao Boey, Charis Jia Yan Soon, Paul J. Gallagher

**Affiliations:** 1 Department of Pharmacy and Pharmaceutical Sciences, Faculty of Science, National University of Singapore, Block S4A, Level 3, 18 Science Drive 4, 117543 Singapore, Singapore; 2 Department of Curriculum and Instruction, School of Education, Indiana University, 201 N. Rose Avenue, 47405 Bloomington, IN USA; 3 Department of Curriculum and Instruction, School of Education, University of Wisconsin– Madison, 225 N. Mills Street, 53706 Madison, WI USA; 4 Center for Medical Education, Yong Loo Lin School of Medicine, National University of Singapore, 10 Medical Drive, 117597 Singapore, Singapore; 5 Department of Pharmacy, Khoo Teck Puat Hospital, 90 Yishun Central, 768828 Singapore, Singapore

**Keywords:** Empathy, Narrative medicine, Narratives, Pharmacy education, Pharmacist, Pedagogical design, Pedagogical evaluation, Pedagogical enhancements

## Abstract

**Background:**

Narrative medicine demonstrated positive impact on empathy in medicine and nursing students. However, this pedagogical approach had not been evaluated in pharmacy education. This study sought to apply and evaluate the narrative medicine approach in extending empathy in Asian undergraduate pharmacy students.

**Methods:**

Narrative medicine was applied through workshops which used narratives of people with different experiences and perspectives. First-year undergraduate pharmacy students who volunteered and attended these workshops formed the intervention group (*N* = 31) and the remaining first-year cohort formed the control group (*N* = 112). A sequential explanatory mixed methods approach was adopted in which quantitative methods were first used to measure impact on pharmacy students’ empathy using the Jefferson Scale of Empathy– Health Professions Student (JSE-HPS), and qualitative methods (i.e. group interviews) were then used to assess pharmacy students’ emotional responses to narratives, and the perspectives of pharmacy students and faculty of this pedagogical approach.

**Results:**

There was no difference in JSE-HPS scores between intervention and control groups across baseline (i.e. upon matriculation), pre-intervention, and post-intervention timepoints. Pharmacy students in the intervention group had lower scores in Factor 3 (“Standing in People’s Shoes”) following the intervention. Five themes, guided by internal and external factors in cognition, emerged from the Group Interviews: (1) incongruence between students’ motivation and faculty’s perception, (2) learning context, (3) academic context, (4) cognitive system, and (5) affective system. Themes 1, 4 and 5 referred to internal factors such as students’ motivation, perceived learnings, and feelings. Themes 2 and 3 referred to external factors including workshop materials, activities, content, and facilitation.

**Conclusion:**

This study is the first to demonstrate that pharmacy students engaged with the narrative medicine approach as narratives elicited emotional responses, exposed them to diverse perspectives, and deepened their appreciation of the importance of empathy and complexities of understanding patients’ perspectives. Scaffolded educational interventions using narratives and real-life patient encounters, alongside longitudinal measurements of empathy, are necessary to bring about meaningful and sustained improvements in empathy.

**Supplementary Information:**

The online version contains supplementary material available at 10.1186/s12909-024-05254-z.

## Background

Empathy is a cognitive attribute that involves understanding others’ experiences, concerns and perspectives, combined with an ability to communicate this understanding and an intention to help [1]. Healthcare providers displaying empathy forged stronger trust and elicited greater disclosures, which led to more accurate diagnosis, improved patient understanding, increased adherence, and better patient outcomes [2–4]. Empathy towards oneself and co-workers was also associated with reduced burnout, increased job satisfaction, and enhanced teamwork [5–8]. Thus, empathy is a crucial element of health professional education as advocated by organizations such as the Accreditation Council for Pharmacy Education (ACPE) and the Association of American Medical Colleges (AAMC) [9, 10].

Numerous pedagogical approaches had been used to nurture empathy in health professional education, e.g. simulations, role plays, reflective writing, experiential learning, communication skills training [11–13], but none of these approaches had clearly demonstrated superiority. Furthermore, effectiveness of these traditional approaches might arguably be questionable as longitudinal studies revealed that empathy of health professional students remained constant or declined as they progressed through their programs [14–17].

More recently, pedagogical approaches that evoke learners’ emotional responses and thought processes had been suggested to be more effective in nurturing empathy [18]. Narrative medicine, which is described as an approach to medicine, or more broadly healthcare, that employs narrative skills to augment scientific understanding of illnesses, is one such pedagogical approach [19, 20]. Narrative medicine integrates approaches from arts and humanities by using narratives in various forms of visual representations to evoke learners’ emotional responses, promote reflective thinking, and hone their listening and observation skills [20]. Health professional educators employed narrative medicine by exposing learners to patient narratives in the form of videos, movies, novels, audio recordings, poetries, photography, or real-world encounters, and by guiding critical analyses of these narratives through discussions and reflections [21–24]. Although the narrative medicine approach had demonstrated positive impact on empathy in medical and nursing education and learners’ perspectives of this approach had largely been positive [8, 23, 24], severe gaps remain in our knowledge of narrative medicine and its role in nurturing empathy. First, existing literature on narrative medicine largely originated from Western contexts [20] and since sociocultural factors influence how empathy is processed [25], more experiences are needed with how learners engage with narrative medicine in different contexts, especially Asia. Furthermore, unlike rich experiences in medical and nursing education, the application of narrative medicine in pharmacy had been limited to medication counselling and ethics trainings in practicing pharmacists [26, 27]. While narratives had recently been suggested to be useful in pharmacy education [28], it is not known if pharmacy students, who of all health care professionals have a greater scientific aspect to their training, would engage emotionally with such narratives as narrative medicine has not been thoroughly evaluated in pharmacy education [29].

In particular, the pharmacy profession has traditionally been product-focused but the profession has since evolved and pharmacists’ roles have transformed to that of a direct patient care provider [30], and thus empathy is also of paramount importance in pharmacy education [9]. Research in cognitive psychology demonstrated empathy as an innate trait and most individuals are more empathetic towards others who are closer to or like themselves [31]. The role of educators is to extend learners’ empathy towards people and issues that would otherwise seem distant in order to support learners’ intentional acts of benevolence in those distant contexts [32]. Consequently, it is reasonable to infer that pharmacy students enter professional education with a level of empathy reflecting their innate empathy and those developed through previous experiences. The role of pharmacy educators is to employ pedagogical approaches, such as narrative medicine, to extend learners’ empathy towards patients who might have different experiences and perspectives from that of their own.

The objectives of this study are: (1) measure impact of the narrative medicine approach on undergraduate pharmacy students’ empathy, (2) establish if undergraduate pharmacy students would engage with the narrative medicine approach by demonstrating emotional responses to narratives, and (3) assess undergraduate pharmacy students’ and faculty members’ perspectives of this pedagogical approach.

## Methods

### Study design and methodology

This was a prospective cohort study which aimed to apply and evaluate the narrative medicine approach in Asian undergraduate pharmacy students using a sequential explanatory mixed methods approach [33]. Quantitative methods were first used to measure the impact of the narrative medicine workshops on empathy. Qualitative methods were then used to enable deeper interpretations of quantitative results by determining how pharmacy students engaged with the narrative medicine approach and exploring pharmacy students’ and faculty members’ experiences and perspectives of this pedagogical approach.

### Study participants and context

Participants were first-year undergraduate pharmacy students enrolled in Bachelor of Pharmacy (BPharm) (Honors) program at the National University of Singapore (NUS) in academic year (AY) 2021–2022. Undergraduate pharmacy students at NUS are primarily from Southeast Asia, with the vast majority being Singaporeans. BPharm (Honors) is a four-year professional degree program consisting mainly of foundational concepts in basic and applied sciences and introduction to professional identity and skills in the first year, followed by integrated physiological systems-based courses and additional professional identity and skills-based courses in subsequent years [34]. Learning in the BPharm (Honors) program is complemented by an inter-professional Common Curriculum for Healthcare Professional Education (CCHPE), in which pharmacy students learn knowledge and skills that are considered essential pillars in healthcare alongside their peers from NUS medicine, nursing and dentistry [35]. Experiential learning is an integral component of the BPharm (Honors) program and pharmacy students’ first patient encounter begins in Year One semester two. This is an inter-professional learning experience within the CCHPE with a focus on understanding the experiences, perspectives, and social and ecological determinants of health for patients living in the community. Pharmacy students have further opportunities for patient interactions in Years Two-Four in various settings including community pharmacy and acute-care [34].

Narrative medicine was applied in Year One of the BPharm (Honors) program to expose pharmacy students to narratives which would provide foundations for understanding diverse perspectives, listening and observation skills essential for demonstrating empathy. Such understandings and skills would be reinforced and further developed in the CCHPE and the BPharm (Honors) program.

### Application of narrative medicine

Narrative medicine was applied through three in-person workshops, two and a half hours each, in December 2021 which was during the vacation between semesters one (August-November) and two (January-April). The workshops were co-developed by clinical faculty members (ZH, KZY, DSYT, CXRN, PJG) at the NUS Department of Pharmacy and Pharmaceutical Sciences who offered insights in pharmacy education and healthcare, and international collaborators who contributed expertise in instructional design and humanities-based pedagogical approaches (KCB, LCH).

The workshops were designed to extend empathy by exposing participants to narratives from people with experiences and perspectives that differed from that of their own (e.g. patients with physical disabilities or memory loss, caregivers of patients with schizophrenia), and by practicing narrative skills whereby participants listened to and observed verbal and non-verbal communication, and reflected on their own emotional responses through discussion with peers and facilitators. Narratives used were in the format of videos, novel excerpts, and poetries which participants watched or read before or during the workshops (Appendix 1). These were selected given their relevance both to the healthcare and sociocultural contexts of Singapore, and various formats were chosen to cater to different learning styles. The design of the workshops was informed by the following pedagogical principles and instructional practices. Firstly, narratives as concrete representations of others’ lives can be effective for extending empathy in healthcare [36–38]. Secondly, in order to critically process and understand narratives, learners need to respond to the narratives, make connections to prior experiences and engage in discussion [39, 40]. Thirdly, effective instruction requires scaffolding, e.g. providing tools such as graphic organizers support learners in learning more than they could on their own [41, 42].

These narratives formed the basis for workshop activities, including empathetic reading, engaged listening and compassionate interviewing. Participants were provided with worksheets that challenged them to describe the emotions shared in the narratives and to reflect on how these narratives influenced their own perspectives (Appendix 2). Participants first completed these worksheets individually and then used these worksheets to guide their discussions in randomly assigned groups. The workshops were facilitated by LCH and KCB. Thirteen faculty members from NUS Department of Pharmacy and Pharmaceutical Sciences and Yong Loo Lin School of Medicine observed the workshops and shared their practice experiences to offer insights on the relevance of empathy and narrative medicine in healthcare during the final workshop session.

### Measuring empathy

Empathy was measured using the Jefferson Scale of Empathy– Health Professions Student (JSE-HPS). JSE was developed for measuring empathy in the healthcare context and is one of the most widely used instruments in health professional education [43]. There are three versions of JSE designed for measuring empathy in practicing healthcare professionals (JSE-HP), medical students (JSE-S) and other health professional students (JSE-HPS). Validity, reliability, and psychometric properties had been rigorously established and published for all three versions [43–46]. JSE-HPS is a self-administered questionnaire with 20 items, each scored on a seven-point Likert scale (total score: 20–140, higher score corresponds to greater empathy) [44]. It also measures three factor constructs of empathy: (1) Factor 1 “Perspective Taking”, (2) Factor 2 “Compassionate Care”, and (3) Factor 3 “Standing in Patient’s Shoes” [45].

### Study procedure

First-year undergraduate pharmacy students enrolled in the (BPharm) (Honors) program at NUS in AY 2021–2022 were invited to participate in the narrative medicine workshops through an open in-class announcement upon matriculation in August 2021. Participation was voluntary but to incentivise participation, pharmacy students who participated in the workshops earned hours that could be applied toward fulfilling their professional development requirement in a mandatory first-year course. A convenience sample consisting of the first 35 pharmacy students who signed up formed the intervention group as the number of workshop participants was limited by prevailing safe distancing measures and venue capacity restrictions during the COVID-19 pandemic. The remaining first-year pharmacy students (*N* = 132) who did not participate in the workshops constituted the control group. Other than narrative medicine workshops, pharmacy students in both groups underwent identical teaching and learning activities in the curriculum. A total sample size of 167 (intervention group: *N* = 35, control group: *N* = 132) will provide approximately 80% power to detect an effect size of 0.6 [47] at a significance level of 0.05.

JSE-HPS was administered to all first-year pharmacy students at three timepoints: (1) August 2021 (baseline), (2) November 2021 (pre-intervention), and (3) January 2022 (post-intervention). Completion of JSE-HPS was voluntary regardless of participation in the workshops. Measurement in August 2021 established pharmacy students’ empathy levels at baseline upon matriculation. In semester one, they undergo communication skills training during which the importance of empathetic responses in patient-centric communication was introduced. Since such training might affect empathy [12, 48, 49], JSE-HPS was administered again at the end of semester one (November 2021) before the narrative medicine workshops.

Workshop participants were invited to join group interviews (GIs) through open announcements. Three face-to-face semi-structured GIs were conducted between January to February 2022, two for pharmacy students and one for faculty. Two semi-structured interview guides with open exploratory questions were developed by core research team members (ZH and PJG), one for pharmacy students’ GIs and one for faculty GI and were validated over a series of quality checks in consultation with a medical educationalist (SSL). To establish if pharmacy students would engage with the narrative medicine approach, interview questions explored whether narratives and activities in the workshops evoked pharmacy students’ emotional responses. Questions also invited them to explain their rationale for participation, and to share their perspectives on workshop content and facilitation. Questions invited faculty members to describe how they thought pharmacy students engaged during the workshops, and to share their perspectives on the role of narrative medicine in health professional education and in their own teaching. Each GI lasted approximately one hour, were audio-recorded, and were facilitated by the same research assistant (RA) (SNW) to ensure consistency.

### Data analyses

Pharmacy students’ demographics were summarized using descriptive statistics and compared between intervention and control groups using the chi-square test. JSE-HPS scores were computed using recommended algorithm, for total score and scores for Factors 1–3 [44, 45]. Missing data were handled by imputing missing scores through multiple imputation. Total JSE-HPS score and scores for Factors 1–3 were compared between intervention and control groups across three timepoints using mixed ANOVA with gender and age as covariates. For non-normally distributed scores, mixed ANOVA with Aligned Rank Transformation (ART) was used, and post-hoc analysis was conducted by repeating the mixed ANOVA with ART for each pair of datasets [50]. Total JSE-HPS scores were also compared between gender and age groups using the independent samples t-test and one-way ANOVA, respectively. All quantitative analyses were conducted using IBM^®^ SPSS^®^ version 27.0 (IBM^®^ Corporation, Armonk, NY). A p-value of less than 0.05 was considered statistically significant.

Audio recordings from three GIs were verbatim transcribed by two RAs (BMYB and CJYS) and analyzed manually using Braun and Clarke’s thematic analysis with an inductive approach [51, 52]. Two RAs examined the transcripts independently and repeatedly to identify relevant ideas and denoted them with succinct codes. Both RAs then discussed and agreed on codes to be used, with discrepancies resolved through discussion with ZH, PJG and SSL. Internal and external factors in cognition [53] were used as a framework to group similar codes into sub-themes and themes. Rigor of the thematic analysis was upheld through constant review of transcripts for new ideas and to refine existing themes [51, 52]. The constant comparative method was used to compare key findings between student and faculty GIs to construct an in-depth analysis [54]. Qualitative rigor was ensured through member checking during the interviews, clear audit trials, independent transcription, collaborative analysis, thick description of workshops and GI participants, triangulation of qualitative and quantitative results, which addressed credibility, transferability, confirmability and dependability in the Lincoln and Guba’s model of trustworthiness [51, 55].

### Ethical considerations

This study qualified for “exemption” status per the NUS Institutional Review Board and therefore, review and approval were granted by the Ethics Review Committee at NUS Department of Pharmacy and Pharmaceutical Sciences (reference number: PHA-DERC-18).

## Results

Out of 167 first-year undergraduate pharmacy students, 147 students (intervention group: *N* = 35, control group: *N* = 112) who completed at least one JSE-HPS administration were included. Four students in the intervention group were excluded because they did not attend all three workshop sessions. Pharmacy students’ gender and age were comparable between the two groups (Table 1).

**Table 1:** Participant Demographics

	Intervention Group (*N* = 31)	Control Group (*N* = 112)	p-Value
**Gender** Female Male	17 (54.8) 14 (45.2)	67 (59.8) 45 (40.2)	0.682
**Age Group (Years)** < 19 19–21 22–24	2 (6.5) 28 (90.3) 1 (3.2)	10 (8.9) 95 (84.8) 7 (6.3)	0.748

Data shown as N (%)

### Quantitative: pharmacy students’ empathy

At baseline, total JSE-HPS score for pharmacy students in this study was 111.2 ± 10.8, and was similar between intervention and control groups (110.3 ± 9.2 versus 112.0 ± 9.2, *p* = 0.367).

There was no significant interaction between the effect of study group and timepoints on total JSE-HPS scores (*p* = 0.955), indicating that total JSE-HPS scores were comparable between intervention and control groups across baseline, pre-intervention, and post-intervention timepoints. Scores for Factor 1 “Perspective Taking” (*p* = 0.435), and Factor 2 “Compassionate Care” (*p* = 0.138) were also similar between intervention and control groups across the three timepoints. On the contrary, there was significant interaction between study group and timepoints for scores in Factor 3 “Standing in Patient’s Shoes” (*p* = 0.049) (Table 2). Post-hoc analyses revealed that the difference was between baseline and post-intervention timepoints, where pharmacy students in the intervention group had decreased Factor 3 scores from 8.4 ± 2.0 to 7.4 ± 2.4, and those in the control group had increased Factor 3 scores from 8.0 ± 2.5 to 8.5 ± 2.2 (Table 3).

**Table 2:** JSE-HPS Scores at Baseline, Pre-Intervention and Post-Intervention Adjusted for Age and Gender

	Baseline	Pre-Intervention	Post- Intervention	p-Value^a^
**Total Score** Intervention Control	110.3 ± 9.2 112.0 ± 9.2	111.3 ± 10.0 112.9 ± 7.5	110.9 ± 10.5 112.0 ± 8.5	0.955
**Factor 1 Score** Intervention Control	59.3 ± 5.4 59.9 ± 5.0	58.8 ± 6.2 60.4 ± 5.2	58.9 ± 6.4 59.4 ± 5.4	0.435
**Factor 2 Score** Intervention Control	42.4 ± 4.1 43.9 ± 4.2	43.2 ± 4.5 44.0 ± 3.7	44.2 ± 4.4 44.1 ± 3.9	0.138
**Factor 3 Score** Intervention Control	8.4 ± 2.0 8.0 ± 2.5	8.5 ± 2.4 8.5 ± 2.3	7.4 ± 2.4 8.5 ± 2.2	0.049

Data shown as mean ± standard deviation

^a^Interaction between study group x timepoints

**Table 3:** Post-Hoc Analysis of Scores for Factor 3 “Standing in Patient’s Shoes”

	Intervention (*N* = 31)	Control (*N* = 112)	p-Value^a^
Baseline vs. Pre-Intervention Baseline Pre-Intervention	8.4 ± 2.0 8.5 ± 2.4	8.0 ± 2.5 8.5 ± 2.3	0.394
**Pre-Intervention vs. Post-Intervention** Pre-Intervention Post-Intervention	8.5 ± 2.4 7.4 ± 2.4	8.5 ± 2.3 8.5 ± 2.2	0.112
**Baseline vs. Post-Intervention** Baseline Post-Intervention	8.4 ± 2.0 7.4 ± 2.4	8.0 ± 2.5 8.5 ± 2.2	0.015

Data shown as mean ± standard deviation

^a^Interaction between study group x timepoints

Total JSE-HPS scores were comparable between pharmacy students of all age groups, but male pharmacy students had higher scores than their female counterparts at pre-intervention (114.2 ± 8.5 vs. 111.4 ± 7.7, *p* = 0.044) and post-intervention timepoints (113.5 ± 8.9 vs. 110.5 ± 8.8, *p* = 0.045) (Table 4). All results showed stability in sensitivity analyses in which pharmacy students who only attended one or two sessions of the workshop were included in the intervention group and those with missing responses on the JSE-HPS were excluded.

**Table 4:** Total JSE-HPS Scores at Baseline, Pre-Intervention and Post-Intervention Stratified by Gender and Age Group

	Baseline	p-Value	Pre-Intervention	p-Value	Post- Intervention	p-Value
**Gender** Female Male	111.1 ± 9.3 112.3 ± 9.0	0.420	111.4 ± 7.7 114.2 ± 8.5	0.044	110.5 ± 8.8 113.5 ± 8.9	0.045
**Age Group (Years)** < 19 19–21 22–24	112.4 ± 9.1 111.5 ± 9.2 112.0 ± 10.1	0.936	109.8 ± 8.1 112.6 ± 8.0 115.8 ± 9.0	0.262	111.0 ± 7.4 111.9 ± 9.0 109.9 ± 11.4	0.786

Data shown as mean ± standard deviation

### Qualitative: engagement with narrative medicine and perspectives of this pedagogical approach

**Fig. 1:**
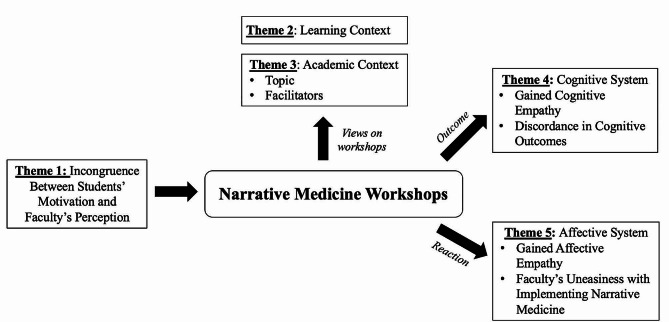
Relationship Between Five Themes from Thematic Analysis

GIs included six pharmacy students (GI 1: *N* = 2, GI 2: *N* = 4) (female: *N* = 3, male: *N* = 3; age < 19 years: *N* = 1, age 19–21 years: *N* = 5) and three faculty members (i.e. GI 3). Five themes emerged and their relationships were outlined in Fig. 1. Themes 1, 4 and 5 referred to internal factors such as students’ motivation, and their perceived learnings and feelings from the workshops. Themes 2 and 3 discussed external factors, e.g. workshop materials, activities, content, and facilitation, that influenced students’ learning.

### Theme 1: incongruence between students’ motivation and faculty’s perception

Since participation in the narrative medicine workshops was voluntary, faculty believed that pharmacy students who participated were intrinsically motived to learn about empathy: *“workshops are also voluntary […] so these are people who are really interested” (F1, GI 3)*. While some students expressed interest: *“I was curious what empathy was all about” (S1, GI 1)*, many identified extrinsic motivators such as fulfilling professional development requirement and giving in to peer pressure: *“this workshop is able to hit 6 hours [of professional development]” (S5, GI 2), “everyone around me was signing up so I was like… okay I guess I am going as well” (S4, GI 2)*.

### Theme 2: learning context

The range of workshop materials (i.e. videos, novel excerpts, poetries) was perceived to be diverse and catered to different learning styles: *“[workshop materials] were good and comprehensive” (S6, GI 2), “students learn in different ways, some can read better, some needs to watch [and some] needs to listen” (F2, GI 3)*. Pharmacy students preferred materials and activities directly related to healthcare. For example, healthcare professional faculty’s sharing of real-life patient stories was well-received because these stories demonstrated the relevance of empathy to students’ future careers: *“because [the sharing is] very interesting and it is like a real-life scenario, […] like someone actually had to deal with this before and I think that helps us see like how it’s applicable to our future” (S5, GI 2)*.

However, cognitive load of workshop materials affected learning. Some materials included medical terminologies and analogies that were confusing to pharmacy students: *“I really don’t understand what’s going on, the words they used were also quite complicated” (S6, GI 2)*. Learning was also affected when pharmacy students were not familiar with their group members: *“I was very annoyed with a group of strangers because it just wasn’t a very focused discussion” (S5, GI 2)*, *“I would prefer [discussing with] my own group [that I know from other courses] because I know how we work and discuss things” (S6, GI 2)*,

### Theme 3: academic context

#### Sub-Theme 3.1: Topic

Pharmacy students and faculty believed that narrative medicine was a useful pedagogical approach because it introduced insightful patient perspectives and exposed learners to the complexities of healthcare: *“it feels like you’re tapping into the unknown […] the kind of stories that you wouldn’t normally hear, so when I first watched it, I found it very enlightening” (S2, GI 1), “I could see that… [the students] connected with the stories well” (F3, GI 3)*, and *“narratives ground students a little bit more because they are so used to… everything is white and black, nothing in between” (F2, GI 3).* Some pharmacy students also felt that the workshops reinforced their baseline understandings: “*the workshop reinforced what I originally felt… the answer [on JSE-HPS] was a very clear strongly agree, so after that it continues to be strongly agree*” *(S6, GI 2).*

#### Sub-Theme 3.2: Facilitators

Pharmacy students and faculty agreed that facilitators for narrative medicine workshops should be those with experiences in healthcare: *“there’s the limitation where the facilitators are not healthcare professionals, they don’t have [healthcare] background, so they cannot go in depth” (S3, GI 2)*”, *“a [healthcare] practitioner would be better because you see patients all the time and you know how to give tips to the students” (F3, GI 3)*.

### Theme 4: cognitive system

#### Sub-theme 4.1: gained cognitive empathy

Narrative medicine workshops were perceived by pharmacy students to improve their cognitive empathy as narratives exposed them to different perspectives and possible reasons for others’ actions: *“[narratives exposed] me to different perspectives of each character and what they’re thinking” (S1, GI 1)*, *“you shouldn’t immediately judge the person… so all these stories actually help build our perception of what could potentially [be] happening” (S2, GI 1)*. Students came to realize the complexities of empathy and the challenges of understanding others’ perspectives: *“empathy is not so simple” (S1, GI 1)*, *“in real life, you actually don’t know the person’s perspective, you only know your own” (S2, GI 1)*. However, students felt that the workshops did not help them apply cognitive empathy towards demonstrating empathetic behaviors: *“[facilitators] said something like first you need to understand, when you understand then you will feel, when you feel very deeply, then it moves you into action, the [workshop] does a good job in the first part but not the second and third” (S2, GI 1).*

#### Sub-theme 4.2: discordance in cognitive outcomes

There also appeared to be a discordance between pharmacy students’ and faculty’s expectations on the cognitive outcomes of the workshops. Students expected the workshops to develop their skills in extending empathy which they felt that the workshops had not been adequate: *“my perception of developing empathy is something like developing the skills required to show empathy, I have the feeling now but whether I am capable enough to use it is another story” (S1, GI 1*). On the contrary, faculty felt that the workshops served as a first step for students to understand empathy. Faculty recognized that demonstrating empathetic behaviors require extensive training, and they did not expect students to do so immediately after the workshops: *“it takes time to develop [empathetic] behaviors… you can’t say that okay, today you learn this, tomorrow I expect you to behave like this” (F1, GI 3).*

### Theme 5: affective system

#### Sub-theme 5.1: gained affective empathy

Pharmacy students and faculty agreed that narrative medicine engaged students’ feelings: “*when I was reading the story I understand and I have the kind of feeling like the compassion, the empathy” (S1, GI 1), “I honestly was very impressed [with the students], thought there was a lot of maturity in their responses” (F3, GI 3*). Pharmacy students reported that narratives evoked emotional responses thereby improving their affective empathy: “*after [watching] the video then [I] felt a bit uncomfortable and also angry” (S2, GI 1), “[in narratives] there’s like descriptive words and it’s just like a reflection of what the person felt so in a way I could set into the shoes of that person” (S4, GI 2*). Nonetheless, many still expressed hesitancies towards empathizing with others out of the fear of misinterpretations: *“sometimes we may think about something that’s not actually what the other person is thinking about, that kind of fear” (S1, GI 1), “I [am] a bit scared to go [in]to like sensitive areas of the person’s life” (S4, GI 2).*

#### Sub-theme 5.2: faculty’s uneasiness with implementing narrative medicine

Faculty expressed significant unease if they were to be tasked with adopting the narrative medicine approach. Health professional educators viewed themselves as science-oriented individuals and therefore, lacking the expertise in narrative medicine: *“we are very science-based people, I won’t say I am completely comfortable teaching” (F1, GI 3*), *“I am scared, I don’t know whether I can achieve the learning outcomes” (F2, GI 3)*.

## Discussion

This study applied and evaluated the narrative medicine approach in Asian undergraduate pharmacy students. Quantitative results demonstrated no significant change in empathy following the narrative medicine workshops. Qualitative results helped rationalize the quantitative findings by suggesting limitations of a one-time intervention (sub-themes 4.1 and 4.2), pharmacy students realizing the complexities of empathy (sub-theme 4.1) and potential ceiling effect with the JSE-HPS (sub-theme 3.1). This study is the first to demonstrate that pharmacy students engaged with the narrative medicine approach as narratives evoked emotional responses such as discomfort, anger, and fear (sub- theme 5.1), thereby contributing to the evidence of narrative medicine in pharmacy education and training. We believe that these findings should support the wider implementation of the narrative medicine approach in the BPharm (Honors) program and for pharmacy education in an Asian sociocultural context, as findings were derived from learners with comparable demographic profile and included those who were intrinsically and extrinsically motived.

This study demonstrated no significant impact of the narrative medicine workshops in extending empathy of pharmacy students as measured by the JSE-HPS. Contrary to our findings, numerous previous studies demonstrated positive impact of narrative medicine on learners’ empathy but among these studies, those conducted in Western populations far exceeded those from other contexts [20]. This study was conducted in Asian students and sociocultural differences might have contributed to disparate results. It had been suggested that Westerners tend to use affective processes to demonstrate empathy, whereas Easterners tend to use cognitive mechanisms [25]. This could mean that Asian students might also learn and process empathy differently from their Western counterparts. Little is known about the optimal pedagogical approaches to extend empathy in different sociocultural contexts and our study, being the first on narrative medicine in Asian pharmacy students, could serve as useful incipience for future research.

Secondly, sustained effects of educational interventions on empathy should be considered. Existing literature often demonstrated improvements in learners’ empathy immediately following an intervention [56–58], but studies demonstrating sustained impact were scant [59]. Many studies revealed no sustained improvements [56, 57], with effects declining as early as seven days post-intervention [56]. In this study, JSE-HPS was administered one month after the narrative medicine workshops, making it possible that any effects from the intervention could have declined by then. Although Kagawa et al. recently reported that narratives improved medical students’ empathy up to six months post-intervention, this sustained effect was only observed among students who underwent an additional “clinical practice orientation” during the six-month follow-up period. Since the orientation focused on attitudes required of student physicians, the authors postulated that it could have affected students’ empathy and advocated for repeated educational interventions for sustainable changes in empathy [60], similar to how our faculty felt that students would require extensive training to develop empathetic behaviors (sub-theme 4.2). Pharmacy students in this study might have expected this one-time intervention to develop their skills in demonstrating empathy (sub-themes 4.1 and 4.2), highlighting the importance for educators to clearly communicate learning outcomes and establish realistic expectations.

Moreover, first-year pharmacy students in this study had high JSE-HPS scores at baseline which was not surprising based on previous literature [61–63]. There is a possibility that subtle changes in empathy might not be detected by the JSE-HPS in the setting of high baseline scores [63]. This ceiling effect also surfaced during the GIs where a student described how response on JSE-HPS was “strongly agree” before the workshops and therefore, could not be increased any further (sub-theme 3.1).

Interestingly, Factor 3 (“Standing in Patient’s Shoes”) scores decreased after the narrative medicine workshops for the intervention group, which might not be unexpected since we know from synthesizing quantitative and qualitative findings that narratives made pharmacy students realize the complexities of empathy (sub-theme 4.1). Particularly, pharmacy students in this study were relatively young and in early stages of their professional training, so they might have limited awareness of diverse perspectives and could have over-estimated themselves at baseline. Lim et al. observed a similar decline in Factor 3 scores in physiotherapy students of comparable age, and also attributed it to students lacking exposure to different perspectives at baseline [64]. Since JSE-HPS is a self-administered instrument, it could also be possible that variations in scores reflected fluctuations in self-calibration instead of actual decline in empathy [65].

Notably, male pharmacy students scored higher than their female counterparts on the pre- and post-intervention JSE-HPS administrations, contrary to previous literature which largely suggested that females were more empathetic than males [46, 56, 61]. We postulated that this difference might be attributed to mandatory military enlistment for male Singapore citizens prior to university enrolment, where “Care for Soldiers” is emphasized as a core value of the Singapore military [66]. Varied experiences and hardships during military training could have also instilled a greater sense of empathy in our male students [67]. Similar findings were found in other studies from Singapore and other jurisdictions requiring male conscription [48, 62, 68].

This study also informed how application of the narrative medicine approach could be optimized. While faculty members felt that catering to different learning styles was useful, pharmacy students did not express a clear preference for narratives in any format based on their learning styles. Rather, some videos were challenging because they included medical terminologies unfamiliar to first-year students (theme 2). This finding serves to guide educators on the selection of narratives which should go beyond catering to different learning styles, and instead educators should focus on aligning content of narratives with where students are in their training. This finding is supported by Newton et al. who found little evidence demonstrating that catering to students’ learning styles truly improved learning [69], and is also consistent with the cognitive load theory which suggests that instructional materials that account for learners’ level of expertise optimize learning [70]. Additionally, consistent with the self-determination theory [71], pharmacy students were motivated to learn when they could appreciate relatedness of content to their future careers (theme 2). Thus, educators applying the narrative medicine approach should make explicit connections between narratives and real-life scenarios in healthcare.

Some pharmacy students expressed frustration in having to discuss emotions with peers that they were unfamiliar with (theme 2). This might be related to restrictions during the COVID-19 pandemic which necessitated mostly online classes in AY 2021–2022, resulting in pharmacy students having little opportunities to interact with peers outside of their team-based learning (TBL) groups that they were assigned to work within for all pharmacy courses. However, not all members of a TBL group would have signed up for the workshops and thus, workshop groups were randomly assigned among participants. Our finding reminds educators of the importance of supporting learners in developing interpersonal relationships and creating a sense of community to support effective learning [72].

Our findings also suggested that faculty members appreciated the value of narrative medicine in extending empathy (sub-theme 3.1) but did not feel confident applying this approach (sub-theme 5.2). To support wider curricular implementation, faculty members with experiences in healthcare and pharmacy practice will need to be trained in narrative medicine and its specific facilitation skills, e.g. textual interpretation, attentive listing and appreciative inquiry, to best support students’ learning [73].

Several limitations could be identified in this study. Firstly, intervention in this study was a one-time occurrence which might be argued to be insufficient to impact empathy. Positive studies often implemented their interventions over several months [56, 59, 74] or longitudinally as part of a curriculum [75], whereas several studies adopting one-time interventions demonstrated no significant impact [76–78]. However, given the paucity of evidence in pharmacy education, we first sought to establish that pharmacy students would engage with the narrative medicine approach through a one-time intervention, and to gain experiences and feedback that would facilitate wider implementation. Secondly, we adopted convenience sampling and could not rule out the possibility that pharmacy students who volunteered for the workshops or GIs might be more receptive to educational innovations or hold particularly strong opinions. Even though age, gender, and baseline JSE-HPS scores were comparable between intervention and control groups, findings might still be confounded by other unmeasured differences. Additionally, the small sample size in the intervention group and a larger control group limited statistical power and the ability to detect significant changes in JSE-HPS scores. Excluding four students who did not attend all three workshop sessions meant that the intervention group was smaller than the minimum sample size. However, we were unable to expand recruitment and account for attrition given prevailing safe distancing measures and venue capacity restrictions at the university in December 2021. The limited number of participants who signed up for the GIs would also mean that we did not reach data saturation and there could be other uncaptured perspectives. Lastly, our study focused on first-year pharmacy students and could not rule out the possibility that more mature learners, particularly those with extensive clinical experiences, might have different perspectives of the narrative medicine approach.

Nonetheless, this study is significant because through qualitative results, it is the first to establish that pharmacy students are receptive to and engaged with the narrative medicine approach, and provides evidence to support and improve the use of narratives in pharmacy education as recently advocated by some educators [28]. Contrary to many previous studies on empathy in pharmacy education which used self-developed instruments to measure empathy [29], this study measured empathy quantitatively using the JSE-HPS which is a validated instrument available in many languages [43], thereby creating future opportunities to compare our results with that from other educators. Furthermore, integration of qualitative and quantitative findings provided us with deeper understanding of students’ learning experiences and ways to optimize learning.

## Conclusions

This study is the first to apply and evaluate the narrative medicine approach in pharmacy education and demonstrated that pharmacy students engaged with the narrative medicine approach as narratives evoked emotional responses, exposed them to diverse perspectives, deepened their appreciation of the importance of empathy and the complexities of understanding patients’ perspectives. These findings enriched the limited body of evidence on the use of the narrative medicine approach in pharmacy education, particularly in the Asian context, and established the proof of concept and identified areas of improvements to facilitate the wider implementation of the narrative medicine approach as foundations for extending learners’ empathy in the BPharm (Honors) program. Nonetheless, recognizing that empathy is a complex construct, pharmacy students, as with all health professional students, require scaffolded educational interventions using narratives and real-life patient encounters to bring about meaningful improvements in empathy. Future studies should measure empathy longitudinally to demonstrate the sustained impact of narrative medicine, in conjunction with other educational interventions and experiential learning, in nurturing empathetic healthcare providers.

### Electronic supplementary material

Below is the link to the electronic supplementary material.

Supplementary Material 1

## Data Availability

All data sets and materials are available from the corresponding author upon request.
