# Supplementary material for: Applying narrative medicine to prepare empathetic healthcare providers in undergraduate pharmacy education in Singapore: a mixed methods study
Source: BMC Med Educ. 2024 Mar 15;24:292. doi: 10.1186/s12909-024-05254-z (PMC10943898; doi:10.1186/s12909-024-05254-z)
Supplement: Supplementary file 1 — Supplementary Material 1 [file 12909_2024_5254_MOESM1_ESM.docx]

**Appendix 1: Patient Narratives and Activities in Narrative Medicine Workshops**

***Pre-Workshop 1***

Watch this video and read this novel excerpt. Pay attention to ideas that you find: 1) insightful and compelling, and 2) confusing or challenging.

- TEDx Talks. Narrative Humility: Sayantani DasGupta at TEDxSLC. [YouTube Video]. 2013. Available from: <https://www.youtube.com/watch?v=gZ3ucjmcZwY&t=70s>
- Fadiman A. 1997. The Spirit Catches You and You Fall Down: A Hmong Child, Her American Doctors, and the Collision of Two Cultures. New York, NY: Farrar, Straus and Giroux.

***Workshop 1***

Introduction

- Brief introduction of workshop facilitators, purposes and structure
- Ice-breaker activity: individually, students draw a picture of a pharmacist

Affinity Mapping Exercise

- Question: What were the most compelling ideas addressed in the reading and video?
- Individually, students generate ideas on post-its.
- Small groups: students collaboratively sort ideas into categories and label the categories.
- Whole group debrief: What ideas stood out? What ideas did you find difficult, confusing, or challenging? What are the implications for healthcare professionals?

Brief Interactive Presentation

- Explain key terms, e.g. empathy, compassion, benevolence
- Rationale and evidence for impact and importance for healthcare

Empathetic Reading

- Use “Understanding Others” worksheet to take notes on the first 3 sections of
  - Hallman T. Family Struggles to Save Pianist’s Music Before Alzheimer’s Can Steal It: His Heart, Her Hands. 2017. Available from: <https://www.oregonlive.com/living/page/family_struggles_to_save_music.html>
- Small groups: students discuss the use of subjects’ own words and their importance
- Whole group discussion of how emotions influenced reactions to this reading
- Students individually use the “Thoughts, Questions and Emotions (TQE)” worksheet to jot down thoughts, lingering questions, and emotions about the reading.

Storytelling and Receiving Activity

- Students to divide into pairs: story teller, story receiver
- Story teller: tells a personal story about something important that happened
- Story receiver: pays full attend, notes non-verbal cues, language, story flow, emotions – do not interrupt story teller
- Story receiver: writes their version of the story capturing the essence, reads back to story teller word for word
- Whole group debrief: What are the challenges of listening to and representing the stories of others?

Engaged Listening

- Use “Engaged Listening” worksheet to complete a guided video analysis of
- Our Grandfather Story. Do People with Disabilities Feel Like One of Us? | Can Ask Meh? [YouTube Video]. 2020. Available from: <https://www.youtube.com/watch?v=tlGCsovLo_4&t=2s>
- Students assigned to 1 individual in the video to focus on the words used, stories told, emotions shared, body language, tone of voice and manner of engagement.
- Small groups: compare notes, discuss significance, consider what’s being said that’s not being said.
- Whole group debrief: return to categories from “Understanding Others” worksheet (i.e. values, attitudes, beliefs, challenges and opportunities, agency, and information needed)

***Pre-Workshop 2***

Watch this video and read this short article. Pay attention to ideas that you find: 1) insightful and compelling, and 2) confusing or challenging.

- TEDx Talks. Honoring the stories of illness | Dr. Rita Charon | TEDxAtlanta [YouTube Video]. 2011. Available from: <https://www.youtube.com/watch?v=24kHX2HtU3o>
- Weinberg RB. 1995. Communion. Ann Intern Med.123(10):804-5.

***Workshop 2***

Affinity Mapping Exercise

- Question: What were the most compelling ideas addressed in the reading and video?
- Individually, students generate ideas on post-its.
- Small groups: students collaboratively sort ideas into categories and label the categories.
- Whole group debrief: What ideas stood out? What ideas did you find difficult, confusing, or challenging? How does the reading reflect Charon’s ideas about honoring the stories of illness? What implications do you think there are for healthcare professionals?

Empathetic Reading

- Use “Understanding Others” worksheet to take notes on excerpts in Chapters 1 and 4 of
  - Lim D. 2016. The Sound of SCH. Singapore: Ethos Books. *(note: this is a story told by a Singaporean author who grew up witnessing her uncle’s long struggle with schizophrenia and her mother’s difficult role as caregiver)*
- Students individually write down responses to the following questions with regard to (1) the author’s uncle, (2) the author’s mother, and (3) the medical professionals. Consider how their beliefs and actions diverge or overlap with each other. Then discuss responses with a partner.
  - What are their values, attitudes, beliefs, or priorities?
  - What are their goals?
  - What are their experiences?
  - How do they try to exert control over their world? What choices do they make?
- Whole group discussion: What are the explanatory models of illness found in the story? How do these different models affect patient care in Singapore and why are they important?
- Students individually use the “Thoughts, Questions and Emotions (TQE)” worksheet to jot down thoughts, lingering questions, and emotions about the reading.

Engaged Listening

- Use “Engaged Listening” worksheet to complete a guided video analysis of
  - What Comes After Life in Prison? Ex-Offenders Answer | Can Ask Meh? [YouTube Video]. 2019. Available from: <https://www.youtube.com/watch?v=DU8urjtHJr0>
- Students assigned to 1 individual in the video to focus on words used, stories told, emotions shared, body language, tone of voice and manner of engagement.
- Small groups: compare notes, discuss significance, consider what’s being said that’s not being said.
- Whole group debrief: What makes these experiences more difficult to understand or interpret? What implications does this have for healthcare?

***Pre-Workshop 3***

Compassionate Interviewing

- Interview an elderly person of your acquaintance using the guidelines provided
- Complete the reflective writing worksheet

***Workshop 3***

Small Group and Whole Group Discussion of the Interview Process

- 3-2-1 task: 3 things that went well, 2 things that didn’t, 1 thing you would do differently next time
- How does this interview expand your understanding of others’ lives? What implications does this experience have for your future healthcare practice?
- How would your interview differ for someone more different than yourself (e.g. someone you didn’t know, someone from a different cultural background)?

Sharing by Faculty Members

- Narratives from their own practice experiences to offer insights on the relevance of narrative medicine in healthcare

Empathetic Reading

- Complete the “Empathetic Reading Exercise” worksheet to take notes on
  - Dent T. The Moon and the Yew Tree. Available from: <https://www.poetryfoundation.org/poems/58441/the-moon-and-the-yew-tree-56d23ccfed82f> *(note: the author was diagnosed with HIV at age 30 and this poem describes the physical and emotional realities of living with HIV. This poem adopts Sylvia Plath’s title “The Moon and the Yew Tree” and situates Plath’s poem with Dent’s own framework)*
- Whole group discussion: What kind of story is the poet trying to tell? What kinds of emotions are communicated in the poem? Identify words and phrases that help convey these ideas and perspectives.

Final Summary and Synthesis

- Individual, small group and whole group reflection on the nature of empathy and compassion, the challenges and significance, and implications for healthcare.

**Appendix 2. Worksheets Used in Narrative Medicine Workshops**

**Understanding Others**

| **Values, attitudes, beliefs**   - What is important to them? - What are their goals? - What would they like to accomplish? | **Challenge and opportunities**   - What are their circumstances (social, material, spatial)? - What are their experiences (what happens to them)? |
| --- | --- |
| **Agency**   - How do they try to exert control over their world? - What choices do they make? | **Information needed**   - What do you wish you knew but don’t? - How might you be misinterpreting this person’s life? |

**TQE Exercise**

| **THOUGHTS**  Write down any thoughts you have after reading and discussing the passage |  |
| --- | --- |
| **QUESTIONS**  Write down any lingering questions you have after reading and discussing the passage |  |
| **EMOTIONS**  Describe your emotions at this point |  |

**Engaged Listening**

**As you watch the video, take notes on …**

| **The words they use that seem especially important** | **The stories they tell and their meaning to them** |
| --- | --- |
| **The emotions they share** | **Their body language, tone of voice, and manner of engagement** |

**Reflective Writing Exercise**

**Interview an elderly person of your acquaintance using the guidelines provided.**

| Try to understand the person’s life from their own perspective, without imposing your own values or frame of references:  **Values, attitudes, beliefs**   - What is important to them? - What are their goals? - What would they like to accomplish?   **Challenge and opportunities**   - What are their circumstances (social, material, spatial)? - What are their experiences (what happens to them)?   **Agency**   - How do they try to exert control over their world? - What choices do they make? | Complete the following from the perspective of the person you interviewed. Try to write a complete paragraph (rather than just a sentence) about each (at least 250-300 words total).  I am…  I wish…  The problem is…, but on the bright side…  I’m trying to… |
| --- | --- |
| Now think about your own perspective, and how it may limit your understanding | Complete the following from your own perspective:  I would like to know more about… because….  I may be misinterpreting this person’s life by … |

**Empathetic Reading Exercise**

**Read this poem aloud to yourself: *Tory Dent—The Moon and the Yew Tree***

| **What are the main themes of the poem?**  Identify the imagery, metaphors, words, and phrases in the poem that help convey these ideas and perspectives. |  |
| --- | --- |
| **What feelings or emotions is the poet trying to convey?**  Identify the imagery, metaphors, words, and phrases in the poem that help convey these ideas and perspectives. |  |
| **Choose the most powerful word or short phrase in the poem and explain why this is meaningful to you.** |  |
| **Now think about your own perspective, and how it may limit your understanding** | Complete the following from your own perspective:  I would like to know more about… because….  I may be misinterpreting this person’s life by … |

**Appendix 3. Analysis of Total JSE-HPS Scores at Each Timepoint by Gender and Age**

**Table A1. Total JSE-HPS Scores at Baseline, Pre-Intervention and Post-Intervention**

|  | **Baseline** | **p-Value** | **Pre-Intervention** | **p-Value** | **Post- Intervention** | **p-Value** |
| --- | --- | --- | --- | --- | --- | --- |
| **Gender**  Female  Male | 111.1 ± 9.3  112.3 ± 9.0 | 0.420^a^ | 111.4 ± 7.7  114.2 ± 8.5 | 0.044^a^ | 110.5 ± 8.8  113.5 ± 8.9 | 0.045^a^ |
| **Age Group (Years)**  < 19  19-21  22-24 | 112.4 ± 9.1  111.5 ± 9.2  112.0 ± 10.1 | 0.936^b^ | 109.8 ± 8.1  112.6 ± 8.0  115.8 ± 9.0 | 0.262^b^ | 111.0 ± 7.4  111.9 ± 9.0  109.9 ± 11.4 | 0.786^b^ |

Data shown as mean ± standard deviation

^a^Total JSE-HPS scores at each timepoint were compared according to gender using independent samples t-test.

^b^Total JSE-HPS scores at each timepoint were compared according to age using the one-way analysis of variance.
